# Quantifying ultrasonic mouse vocalizations using acoustic analysis in a supervised statistical machine learning framework

**DOI:** 10.1038/s41598-019-44221-3

**Published:** 2019-05-30

**Authors:** Adam P. Vogel, Athanasios Tsanas, Maria Luisa Scattoni

**Affiliations:** 1 Centre for Neuroscience of Speech, The University of Melbourne, Victoria, Australia; 2 Department of Neurodegeneration, Hertie Institute for Clinical Brain Research, University of Tübingen, Tübingen, Germany; 3 Redenlab, Victoria, Australia; 4 Usher Institute of Population Health Sciences and Informatics, Medical School, University of Edinburgh, Edinburgh, UK; 5 Oxford Centre for Industrial and Applied Mathematics, Mathematical Institute, University of Oxford, Oxford, UK; 6 Research Coordination and Support Service, Istituto Superiore di Sanità, Rome, Italy

**Keywords:** Behavioural methods, Social behaviour, Diagnostic markers, Neurological manifestations

## Abstract

Examination of rodent vocalizations in experimental conditions can yield valuable insights into how disease manifests and progresses over time. It can also be used as an index of social interest, motivation, emotional development or motor function depending on the animal model under investigation. Most mouse communication is produced in ultrasonic frequencies beyond human hearing. These ultrasonic vocalizations (USV) are typically described and evaluated using expert defined classification of the spectrographic appearance or simplistic acoustic metrics resulting in nine call types. In this study, we aimed to replicate the standard expert-defined call types of communicative vocal behavior in mice by using acoustic analysis to characterize USVs and a principled supervised learning setup. We used four feature selection algorithms to select parsimonious subsets with maximum predictive accuracy, which are then presented into support vector machines (SVM) and random forests (RF). We assessed the resulting models using 10-fold cross-validation with 100 repetitions for statistical confidence and found that a parsimonious subset of 8 acoustic measures presented to RF led to 85% correct out-of-sample classification, replicating the experts’ labels. Acoustic measures can be used by labs to describe USVs and compare data between groups, and provide insight into vocal-behavioral patterns of mice by automating the process on matching the experts’ call types.

## Introduction

Rodent models of disease can replicate key histopathological, biochemical, and behavioral features of human disease. Transgenic and conditional mouse lines allow for the study of disease mechanisms, and play a crucial role in pre-human testing of target therapies. Ultrasonic vocalizations (USVs) produced by rodents are consistent and robust phenomena used as an index of social interest, motivation and emotional development^1^. Specific and deep phenotyping of animal models is a crucial step in experimental biology for several reasons: (1) recording and measuring mouse ultrasonic vocalizations has demonstrated utility in a wide spectrum of disease/disorder contexts including autism^2^, hereditary speech disorder^3^, oxytocin^4^ and pain^5^; (2) USVs have been employed as behavioural markers of disease progression and treatment in neurodegenerative diseases including Parkinson’s disease^6^, Huntington’s disease^7^, and ataxia^8^; (3) the underlying mechanisms used by mice to produce USVs are not well understood and the characteristics of USVs can be better defined; (4) the universality of vocal communication in humans and mice makes it an attractive candidate with strong face validity for experimental models requiring quantitative clinical markers of disease mechanisms or treatment response.

The use of sophisticated acoustic analysis methods for identifying salient features of vocal behavior are well established in human research. Studies describing USVs however, have historically relied on less sophisticated approaches such as the number of calls produced^7,8^, or where acoustic analyses have been applied, a limited number of coarse characteristics describing timing, frequency and intensity^6^. Some methods for classifying USVs seek to describe components of each call based on their spectrographic/sonographic appearance or by cluster analysis/dimensionality of call features^9^. However, the most commonly used methods to classify USVs are described by Holy and Guo^10^, and Scattoni and Crawley *et al*.^11^. This visual approach was developed at the US National Institutes of Mental Health (NIMH) in the Behavioural Neuroscience Section led by Prof. Jacqueline Crawley, in conjunction with leading researchers in the field at the Istituto Superiore di Sanità, Roma (Laura Ricceri and Igor Branchi). Scattoni *et al*.^11^ call categories are based on previous categorization approaches^12–14^. Other methods for example, do not offer additional call types or descriptions but use a paired down or expanded version of the same scheme used in the current study (see list of studies in Supplementary Note III for details).

Existing methods for evaluating USVs are limited by their subjectivity and simplicity. Investigators provide qualitative observations or coarse quantitative descriptions of calls without knowledge of those acoustic measures salient to each call type. An alternative method to subjective user dependent measures, acoustic analysis, offers a principled objective framework to characterize calls on the basis of signal processing algorithms capitalizing on time-domain and spectral information representation. The objective and salient acoustic measures are derived from existing and widely used instrumentation which could in practice enable researchers to directly compare between labs, strains and over time.

Here we report the design and implementation of an acoustic analysis protocol for objectively describing USV call types as determined by expert judgement, in mice. Methods will bring the field of USV phenotyping in line with sophisticated methodologies used in human studies and will provide capacity to objectively monitor change over time in rodent treatment trials.

## Results

**Figure 1:**
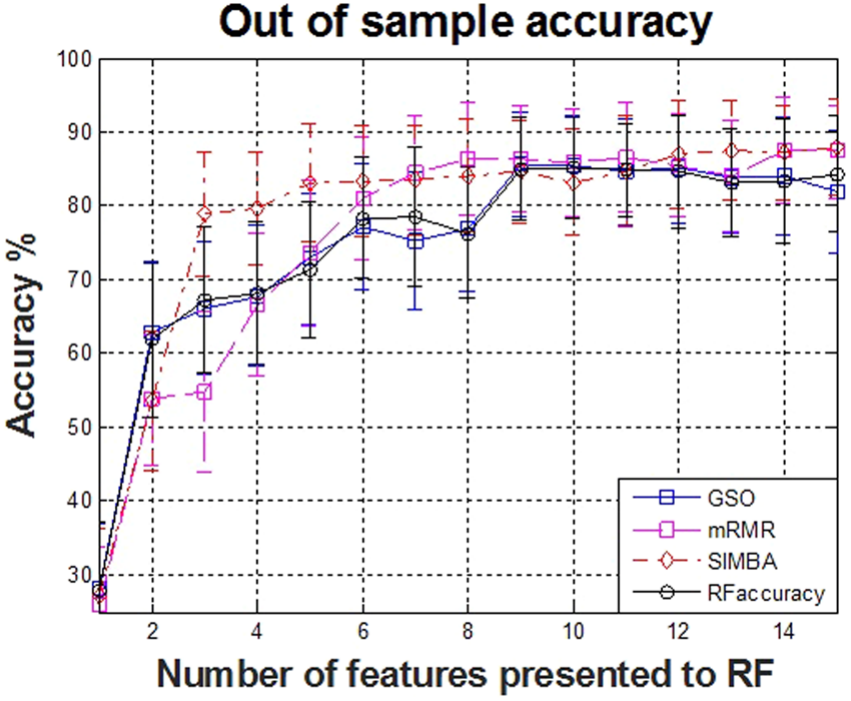
Comparison of out of sample mean performance results with confidence intervals (one standard deviation around the quoted mean performance) using the features selected by each of the four feature selection algorithms. These results are computed using 10-fold cross validation with 100 repetitions. For clarity, we present here only the first 15 steps.

The selected features for each of the four feature selection algorithms appear in Table 1, along with the out of sample accuracy for RF. The features are listed in descending order of selection, aiming at each step to maximize the internal criterion of the corresponding feature selection algorithm. The duration of the phonation, the peak frequency and the maximum and minimum frequencies all appear to be consistently selected amongst the top features by the feature selection algorithms. The out of sample accuracy for predicting the call types as a function of the selected features for each of the feature selection algorithms is summarized in Fig. 1. In agreement with some previous studies^15,16^ focusing on multi-class classification problems with a relatively limited number of samples, we found that RF generally outperformed SVMs; hence, we present results only for RF. Overall, using 8–10 features leads to approximately 85% classification accuracy, and using 15 features leads to 88% classification accuracy using RF and the selected features with SIMBA.

**Table 1:** Selected feature subsets and classification performance.

GSO	mRMR	SIMBA	RFaccuracy
duration	min freq (meanentire)	**duration**	duration
peak freq (stddeventire)	duration	**quart 50 (start)**	peak freq (stddeventire)
max freq (stddeventire)	quart 25 (meanentire)	**peak freq (stddeventire)**	max freq (stddeventire)
quart 50 (stddeventire)	bandw (start)	**peaktopeak**	quart 50 (stddeventire)
min freq (stddeventire)	peak freq (end)	**max freq (stddeventire)**	min freq (stddeventire)
quart 50 (minentire)	peak freq (stddeventire)	**peak freq (start)**	quart 50 (minentire)
quart 25 (mean)	peak freq (start)	**peak freq (end)**	quart 25 (mean)
max freq (minentire)	peak freq (centre)	**quart 75 (start)**	max freq (minentire)
quart 50 (start)	min freq (start)	entropy (mean)	quart 50 (start)
peak freq (start)	fundamental (max)	bandw (stddeventire)	peak freq (start)
peak freq (minentire)	quart 25 (mean)	entropy (maxentire)	peak freq (minentire)
min freq (minentire)	entropy (centre)	bandw (maxentire)	min freq (minentire)
max freq (start)	quart 75 (end)	quart 25 (meanentire)	max freq (start)
quart 25 (meanentire)	max freq (stddeventire)	peak ampl (end)	quart 25 (meanentire)
quart 75 (stddeventire)	quart 25 (end)	min freq (mean)	quart 75 (stddeventire)
82.0 ± 8.3	87.4 ± 6.3	88.0 ± 6.4	84.2 ± 7.4

The last row presents the % accuracy when the selected 15 features from each algorithm are fed into the RF classification algorithm. The results are given in the form mean ± standard deviation and are out of sample computed using 10-fold cross validation with 100 repetitions for statistical confidence. Features are listed in order of selection by the feature selection algorithm. Using only 8 features selected with SIMBA (appearing in bold) we can achieve 85% out-of-sample classification accuracy.

**Figure 2:**
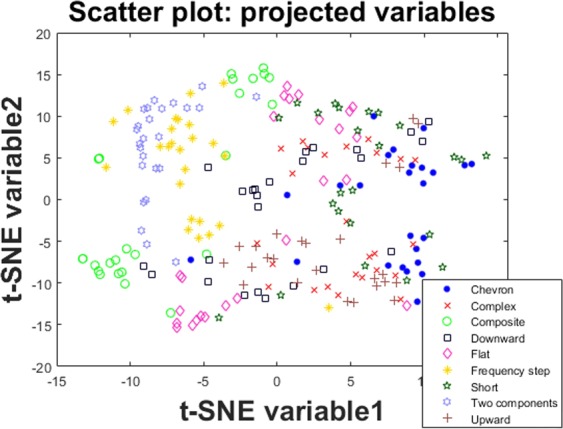
Projecting all 102 features on a 2D scatter plot to visualize the distribution of the classes and how well samples appear to group.

**Figure 3:**
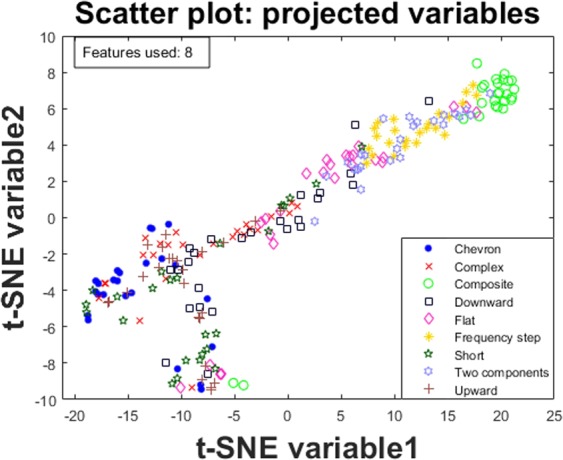
Projecting the top eight selected features using SIMBA on a 2D scatter plot to visualize the distribution of the classes and how well samples appear to group.

Sophisticated tools such as RF and SVM do not provide intuitive visual understanding of the mechanism to obtain the results. To facilitate some insight into the problem we use t-SNE as a standard visualization tool. Projecting the high dimensional feature space in 2D to visualize the differentiation of the USV classes shows how the samples are scattered using all 102 features (Fig. 2); subsequently in Fig. 3 we observe the scatter plot when using a subset of the eight features selected using SIMBA (see Table 1 for the features we used).

Figure 3 highlights that most of the samples belonging to the same class appear to be grouped together; this supports the concept that the features used (and the eight features selected using SIMBA) may be able to differentiate the classes. Since t-SNE is an unsupervised approach (i.e., it makes no use of the expert-provided labels) these figures also indirectly support the expert-provided labels which are used as ground truth in this study in a principled supervised learning paradigm.

## Discussion

We investigated the potential of using acoustic analysis to extract features which can be used to replicate the expert provided labels (9 call types) which represent vocal behavior. We used standard feature selection methods to obtain parsimonious models to decide on a small set of features which has maximal predictive accuracy when fed into state of the art classifiers such as RF and SVM. We demonstrated that a principled statistical machine learning setup with RF using only 8 features selected using SIMBA can provide out-of-sample 85% classification accuracy. In the Supplementary Material we delve deeper in the model’s performance to explore the call types which are correctly detected most of the time and the call types which are occasionally misjudged for a different type by studying the resulting confusion matrix. Overall, all call types were accurately estimated, in particular “Frequency step”, “Short”, and “Two components”. The two class types which appear to be somewhat less accurately estimated were “Chevron” and “Downward”. To our knowledge, this is the first time that a system is developed to characterize USVs and then assign them into call types by means of a principled supervised learning framework to match the experts’ labels. Other standardized systems in this domain such as the Avisoft SAS Lab Pro Recording and the Noldus Ultravox do not currently provide mapping of the extracted features onto expert-labelled call types which are used as the standard in this field to describe vocal behaviour.

**Figure 4:**
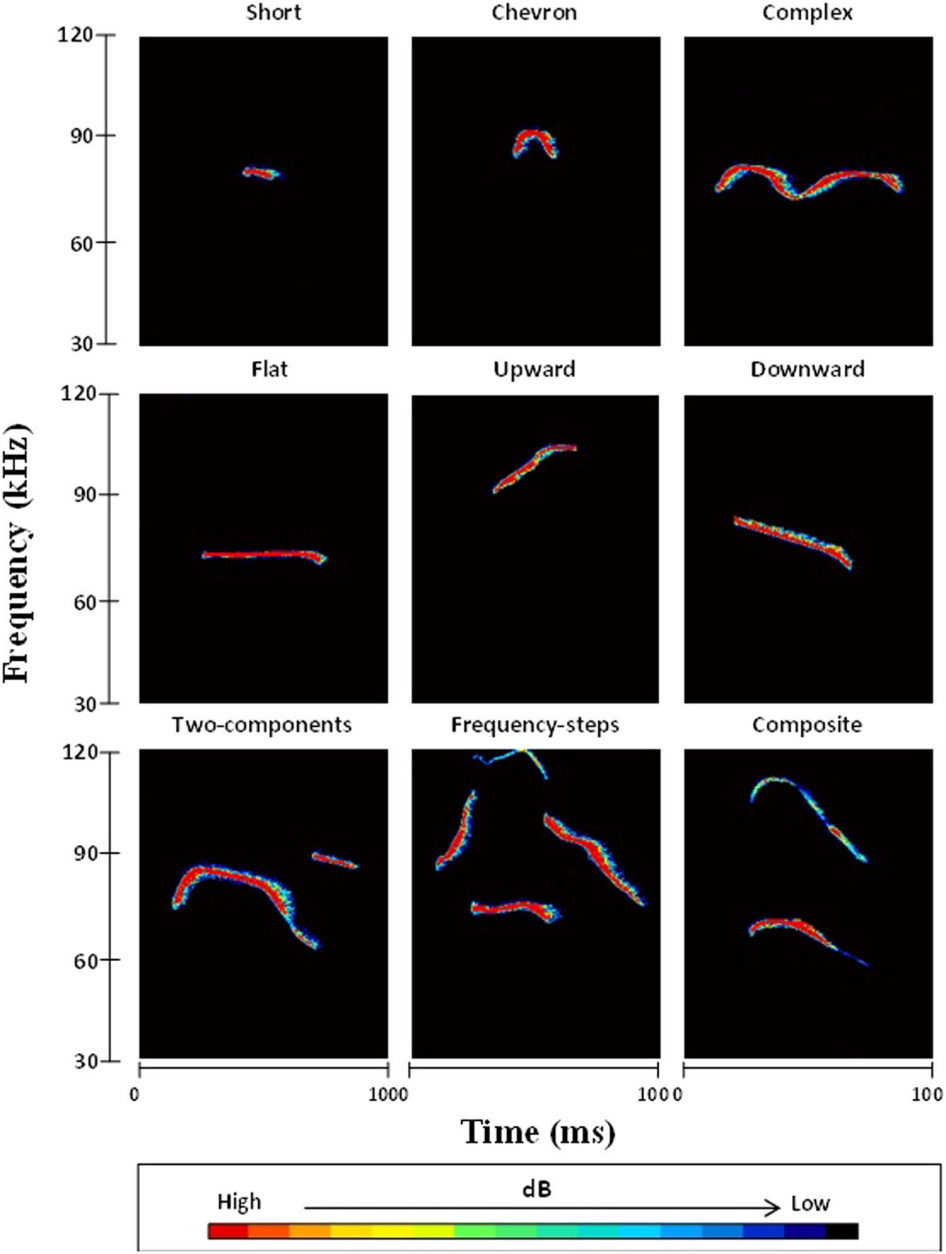
Typical indicative sonograms of ultrasonic vocalizations of mouse pups illustrating nine distinct call categories. From left to right and from top to bottom: ultrasonic vocalization representing *short, chevron, complex, flat, upward, downward, two-components, frequency steps and composite* call categories. Time (in milliseconds) is indicated by the X-axis, frequency (in kHz) is indicated by the Y-axis. *Intensity data (as shown in color) is indicative of relative values and does not represent real intensity outcomes.

We remark that any supervised learning setup aims to replicate some expert-defined labels which are used as the ‘ground truth’. In many fields, there is often some controversy regarding what constitutes the gold standard when it comes to human assessment. Here, we used as ground truth the widely accepted labels proposed by Scattoni *et al*.^11^ which have been used by many different groups for assigning USVs into one of the nine call types (see Fig. 4). The fact that RF accurately replicate the call types supports the notion that the provided labels have solid basis because there are observable traits of the data that are extractable by both humans and machines. It is important to note, the methods used in this study attempted to replicate the expert determined labels as a means of assessing vocal behaviour and not as the ‘de facto’ optimal call type categorization scheme.

We used four established FS algorithms to determine robust, parsimonious feature subsets (see Table 1). There is considerable overlap in the top selected features across all four approaches, giving confidence that these features are critical for replicating the expert call types. For example, the duration, the peak frequency (stddeventire), and the max frequency (stddeventire) are consistently chosen amongst the very top. It is possible to then tentatively interpret how these features assist in class differentiation: e.g. the feature ‘duration’ differentiates the call types ‘short’ and ‘chevron’. Similarly, by inspecting Fig. 4 we observe that the peak frequency can differentiate well some call types. It is the combination of these traits in the supervised learning algorithm which gives rise to accurately determining the call type. We found that SIMBA was superior to the competing FS algorithms in terms of the RF classification accuracy, particularly in the first few selected features (see Fig. 1). This possibly indicates that *complementarity* (joint association of features with the outcome) is critical in this application, a finding which we have previously observed when studying human phonations^16^.

Despite the popularity of powerful tools such as RF and SVM, there is little intuitive understanding into how and why they work well for a particular problem. Here, we used the well-known t-SNE algorithm to project the high dimensional data into 2D and visualize how the entire feature space (102 features) and the selected feature space (5 features) appear in a scatter plot where the call types are marked. Intuitively, one can visually assess in Figs 2 and 3 how data cluster together for the different call types, which provides some justification regarding the success of the supervised learning algorithms.

The underlying mechanisms driving neuromuscular control of mice to produce USVs are not well understood. Theories range from calls produced by oscillation of superficial vocal fold layers, with reduced mass and increased tension compared to true vocal folds^17,18^, a hole tone whistle mechanism^19^ similar to a whistle, or more recently via intra-laryngeal planar impinging jets relating to flow structures from the glottis and filtering within the vocal tract^20^. The acoustic properties of USVs are also poorly described, and it has been suggested that differences between call types are the result of inter-rodent differences in laryngeal structures and neuromuscular planning and execution^20^, as is the case in other vocalizing mammals including humans. Mouse calls produced at frequencies closer to the range of human hearing (e.g., <20 kHz) are known to contain harmonics or formants similar to human vowels^21^ yet the acoustic characteristics of USVs remain unclear. Objective analysis of human based communication can be achieved using automated methods, even when describing large datasets^22^, and methods for defining the rodent equivalent are needed.

The small number of salient acoustic features determined through feature selection in this study objectively describes the acoustic profile of the call types. For animals to act as appropriate experimental surrogates in models of human disease they need to exemplify valid endophenotypes of each disorder. Where communicative breakdowns occur in the human form of the disease, an analogous breakdown could be anticipated in the concomitant mouse phenotype. Refinement and quantification of methods to measure mice vocal behaviors extends our capacity to accurately compare intra-study inter/intra-mouse performance. It also gives rise to the possibility of linking the communicative phenotypes of humans and mice with comparable metrics.

The heterogeneity of call types is one of the barriers to successful automated analysis. If a method is to only choose one representative sample rather than a wide range of calls that fit within the standard characterisation protocol used within the field, they may be neglecting key components of the communicative profile of the animal. The proposed method differs to existing protocols as we have applied multiple forms of the one call type, as determined by multiple raters. This method ensures that the intra-call type heterogeneity observed in mouse USV experiments is catered for by the variance within the sample of 25 calls (albeit derived from pups only, not adult mice). By incorporating multiple calls from a variety of mice (e.g. pup and adult mice), we can justify statistical exploration of features, rather than basing our assumptions on a single animal within a single recording. It also ensures that we avoid a common problem in clinical and medical investigations, that is, trying to change the standard, industry accepted norms by describing data in an alternate way. For example, recent efforts to apply acoustic and clustering techniques for classifying USVs are described by Burkett *et al*.^9^. They employed semi-automated analysis and clustering algorithms using a significantly smaller battery of acoustic measures than applied in the current study. Where they had calls that did not fit into a single category, calls were allocated to “miscellaneous”, “double” and “triple” classes. Burkett *et al*.^9^ tried to fit acoustic components/clusters into visually determined call types and attempted to verify how well they believed the ‘ground truth’ was actually representative of calls.

Future USV analysis developments may simultaneously combine observable motor behaviour (e.g., standing/sniffing) with acoustic measures of vocalization to create an index of communicative behaviour. USV analysis already enables characterization of call types based on the visual features of the waveform, and study of footage of rodent interactions can provide data on social competence. In the interim, we have provided a rationale, detailed methods and evidence of sensitivity/suitability of sophisticated acoustic measures to describe mice USV. The next step is to apply these methods in a variety of strains and disease models.

## Methods

### Experimental animals

C57BL/6J mice, originally purchased from The Jackson Laboratories (Bar Harbor, ME) were bred in our animal facility at Istituto Superiore di Sanità in Rome. Trios (two females and one male) were housed in (33 cm × 13 cm × 14 cm) standard Plexiglas boxes, with sawdust bedding and a metal top. Mice were maintained on a reversed 12:12 h light: dark cycle (lights on at 19:00 h). The temperature was maintained at 21 ± 1 °C, relative humidity (60 ± 10%). Ten days after pairing for breeding, pregnant females were individually housed and checked daily for delivery. At pnd 21, pups were weaned into cages of same sex pairs (3–4 in each cage) and provided with water and pellet (Mucedola, Settimo Milanese, Italy) ad libitum. Recordings have been performed at pnd 8 during the dark phase of the cycle (between 9.30 and 14.00). All procedures were conducted in accordance with the European Communities Council Directive (2014/26 EU) and formally approved by Italian Ministry of Health.

### Ultrasonic vocalization elicitation paradigms

Calls were drawn from protocols described in Scattoni *et al*.^11^ and Romano *et al*.^23^. At pnd 8, one male from each litter (for a total of 25 litters) was selected for the three-minute-session of ultrasonic vocalizations recording. The apparatus consists in an Ultrasound Microphone placed over an empty plexiglass cup (diameter 5 cm and height 10 cm) located inside a sound-attenuating Styrofoam box. Each pup was weighed and its axillary temperature measured after the recording session and immediately placed back in the cage with its mother and siblings. We selected one call per type from each three-minute-wav file.

### Vocalization recording equipment

An Ultrasound Microphone (Avisoft UltraSoundGate condenser microphone capsule CM16, Avisoft Bioacoustics, Berlin, Germany) with a relatively flat frequency response was used to record USVs (see http://www.avisoft.com/usg/cm16_cmpa.htm for microphone specifications). The microphone is sensitive to frequencies between 10 and 250 kHz and was fixed above the cage. The microphone was coupled with an UltraSoundGate 116 H recording interface with a USB 2.0 connector linking to a laptop PC using Avisoft-RECORDER USGH software (Version 3.2) (see http://www.avisoft.com/usg/usg116h.htm for hardware specifications). The sampling rate for the data was set to 250 kHz.

### Qualitative USV analyses

Twenty-five examples of each call type described by Scattoni *et al*.^11,23^ were identified from spectrograms of USVs. The call types are: (1) complex, (2) two components, (3) upward, (4) downward, (5) chevron, (6) short, (7) composite, (8) frequency step, and (9) flat (see Fig. 4 for indicative spectrum constellation of the call types).

Calls were allocated to one of the nine predefined call categories by an expert rater and verified by a second independent rater based on criteria stipulated in Scattoni *et al*.^11^. Both raters agreed on call categories. Calls were classified based on changes in pitch, duration, and trajectory using our previously published methods^11^. Specifically, we note the following characteristics for each call class: (1) *Complex* calls included one component containing two or more directional changes in frequency, each ≥6.25 kHz; (2) *Two-component* calls have two parts: a main call (flat or downward) with an additional shorter component at a different frequency towards the end; (3) *Upward* calls are a single harmonic that increases in frequency ≥12.5 kHz from beginning to end; (4) *Downward* calls follow the opposite trajectory to *Upward* calls, with a single harmonic that decreases in frequency ≥12.5 kHz from beginning to end; (5) *Chevron* calls resemble an ‘inverted-U’; (6) *Short* calls are brief single harmonic calls ≤5 ms; (7) *Composite* calls are formed by two harmonically independent components emitted simultaneously at different frequencies; (8) *Frequency step* calls include multiple instantaneous frequency harmonics appearing as a vertically discontinuous “step” on a spectrogram, but with no interruption in time; (9) *Flat* calls are a single constant harmonic at a stable frequency (≤3 kHz change). For access to the corpora used in this study, contact the authors.

### Quantitative acoustic analysis protocols

Acoustic analysis of USVs was performed using Avisoft SASLab Pro (Version 4.40). Calls were saved as individual files to ensure USVs were accurately identified using the automatic parameter measurement function in Avisoft. Calls were identified within Avisoft using thresholds suitable for each call type. For example, calls allocated to the ‘two-components’ category were identified using the automatic threshold (two thresholds) with a hold threshold of 20 ms. Element separation is required where components of the call have different amplitudes. Adjustment of the hold threshold length is necessary where calls may have extended pauses between components (see Table 2 for settings for call types).

**Table 2:** Thresholds used for automated element separation required for automated parameter measurements.

Call type	Detection method
Chevron	Automatic (whistle tracking)
Complex	Automatic (whistle tracking)
Composite	Automatic (two thresholds)
Downward	Automatic (two thresholds)
Flat	Automatic (whistle tracking)
Frequency-steps	Automatic (three thresholds) Hold threshold 25 ms
Short	Automatic (whistle tracking) Min duration 1 ms
Two components	Automatic (two thresholds) Hold threshold 20 ms
Upward	Automatic (two thresholds)

Spectrograms were produced using the following settings: FFT-length of 512 points; frame size of 100%; bandwidth of 635 Hz; time window overlap of 75% (Hamming window); time resolution of time 0.512 ms; frequency resolution of 488 Hz. A high pass filter of 30 kHz was applied to reduce the impact of background noise.

Acoustic measures derived from Avisoft included summative (i.e. mean/entire) and position specific (i.e. start/end of call) measures of duration (e.g. length of call), frequency (maximum frequency), amplitude (standard deviation intensity), quality (e.g. noise to harmonics ratio), and entropy (i.e. disorder or randomness) at different positions along the call. In total, each USV was characterized using 102 acoustic measures (see Supplementary Materials Table 1 for complete list). Therefore, we processed a design matrix of size 225 × 102 which contained no missing values. The USVs were processed in AVISOFT, and the computed acoustic measures were stored in Excel spreadsheets. All the following processing was completed in Matlab, version 2016b.

### Feature selection

The large number of acoustic measures used in this study (102) may lead to over-fitting due to the curse of dimensionality: the feature space will be inadequately populated with only 225 samples. Obtaining a compact, parsimonious feature subset could improve the generalization performance of the classifier (that is, performance in new, unseen data), and offers tentative insight into this application by interpreting the signal properties which are quantified by the most predictive feature set^24^. In principle, an exhaustive search through every possible feature subset combination would give the optimal results, but it is computationally intractable. Instead, we tested four popular well-established feature selection (FS) algorithms to select parsimonious, information-rich feature subsets. Each algorithm relies on different mathematical foundations, and it is impossible to know *a priori* which approach would work best. Nevertheless, if certain features are consistently selected using different FS algorithms, this could inspire confidence regarding their merit in the studied application. The following contains a short summary of the properties of the FS algorithms used in this study, and we refer to the detailed references for further discussion.

The Gram-Schmidt orthogonalization (GSO) is a sequential forward FS algorithm, where a feature is selected at each step on the basis of being maximally correlated to the response and minimally correlated to the existing feature subset. It projects the candidate features for selection at each step onto the null space of those features already selected in previous steps: the feature that is maximally correlated with the target in that projection is selected next. The procedure iterates until the number of desired features has been selected. Further details of the GSO algorithm used for FS can be found in Stoppiglia *et al*.^25^ and Guyon *et al*.^26^.

The minimum redundancy maximum relevance (mRMR)^27^ algorithm aims to simultaneously maximize relevance (association strength of features with the response) and minimize redundancy (association strength between pairs of features). It only accounts for pairwise redundancies and neglects complementarity (joint association of features towards predicting the response), but often worked well in practice. SIMBA is a feature-weighting algorithm, aiming to select features which contribute towards maximizing the separation of samples from different classes^28^. It is conceptually similar to the nearest neighbour concepts which are widely used in machine learning for classification. Compared to the preceding algorithms, SIMBA uses complementarity as an inherent part of the FS process. The last FS algorithm is integrally computed as part of one of the classifiers used in the study (random forests), and is described below.

Each FS algorithm was run 100 times using perturbed versions of the original dataset to assess FS stability and ensure the robustness of the findings, following the methodology we have described previously^29,30^. In brief, for each FS algorithm the feature subset was selected using cross-validation (CV), using only the training data at each CV iteration (90% randomly selected samples at each iteration). The CV process was repeated a total of 100 times, where in each iteration the *M* features in the dataset (*M* = 102) appear in descending order of selection. Subsequently, a voting mechanism determined the order for the ranking of the 102 features.

### Statistical mapping: estimating the response

We studied the functional relationship of the acoustic measures with the class types in a standard supervised learning setup. We used two state of the art statistical mapping algorithms, random forests (RF)^31^, and support vector machines (SVM)^32^. Efficacy of both classifiers are well established in the machine learning practice, with RF being described as the best off-the-shelf classifier^24^.

### Random forests (RF)

RF (also known as ensembles of decision trees) is a collection of multiple *weak learners*, the decision trees, where each of the weak learners is better than chance and contributes in exploring certain regions of the feature space. Each decision tree is grown by recursively finding the best split of a single feature, amongst the range of the candidate features. Starting with the entire dataset, the first split leads to a partition of the feature space on the basis of a dispersion metric (Gini index), resulting in approximately two halves of the original data. Repeating the process for each of the resulting subsets leads to multiple branches which resemble a tree, each time splitting a subset into two further subsets. Essentially, this partitioning process focuses on finer and finer regions of the feature space. The process stops when reaching a region of the space comprising a single sample, and the outcome for that particular hyper-rectangle in the feature space is assigned to the response of the corresponding sample. The output of the RF is derived using majority voting from all trees, where each tree casts a vote for the query sample (in this case a USV for which we would like to query the RF to determine the membership in one of the nine classes). Each tree is constructed using a bootstrapped version of the original dataset (i.e. resampling with replacement the original data), thus introducing the notion of randomness: this along with the use of the trees as weak learners to build the ensemble explain the name of the method. RF has two hyperparameters: (a) the number of trees which will case a vote, and (b) the number of features over which to search for the optimal split in each node. RF has been described as the best off-the-shelf classifier^24^, to a large extent because the choice of the hyperparameters does not affect considerably the performance of the algorithm in unseen data. Given the RF is relatively insensitive to the choice of its hyperparameters^31^, we used the default option according to Breiman setting the number of trees to 500. The minimum number of observations per child node was set to 1, as is standard in classification settings. We experimented using the default search over the square root of the dimensionality of the presented dataset (102 features), i.e. searching over 10 randomly selected features in each decision for the best split of the data, and twice that value (20 features randomly selected each time), in accordance with Breiman’s recommendations^31^. RF internally selects the best candidate features as an integral part of the statistical mapping process. This information can be used as a FS algorithm itself, where features are assigned weights on the basis of how much they improve the classification accuracy. This approach was used as the fourth FS algorithm in the study and is denoted “RFaccuracy”. For further background information on RF we refer to Breiman^31^ and Hastie *et al*.^24^. We used the Matlab RF implementation available at: https://code.google.com/archive/p/randomforest-matlab/.

### Support vector machines (SVM)

SVM is a fundamentally different approach to RF. Conceptually, the aim is to find the optimal decision boundary (separating hyperplane) to maximize the margin between samples belonging to the different classes^9^. Only a few of the original samples are used to define the decision boundary, and these samples are known as *support vectors* (hence the name of the method). Because the data is in practice not linearly separable, it is necessary to use the *kernel trick*, projecting the data from the original feature space into a higher dimensional space where they may be more easily separable. The width of the kernel is a hyperparameter of the SVM which needs to be determined. To ensure the generalization strength of the SVM in new unseen data, the decision boundaries need to be carefully determined in order to avoid overfitting the data used in the training phase: for this reason, a penalty hyperparameter is used. SVM were originally developed as a binary class classifier, and then are extended to multi-class classification settings using either a One-Against-One (OAO) approach, or One-Against-All (OAA) approach. Here, we used the standard suggestion by Lin^33^ (developer of the LIBSVM package) and used the OAO approach to use the SVM directly for the 9-class problem studies here. Contrary to RF, SVM is highly sensitive in the choice of its hyperparameters. We used the LIBSVM implementation^33^ with a radial basis function (RBF) SVM and followed the standard suggestion of searching through a grid space to optimize the penalty hyperparameter (2^−5^, 2^−3^, …2^15^) and the kernel width hyperparameter (2^−15^, … 2^3^). The optimal SVM hyperparameters were determined through an internal 10-fold CV (see following section for details) set selected from the training data in the learning process; once these values were decided, we assessed its generalization performance using the process described below. For further detail on SVM, we refer to Hastie *et al*.^24^.

### Classifier validation and generalization performance

To assess the generalization performance of the classifier, we used 10-fold CV with 100 repetitions for statistical confidence. This is a principled, standard resampling statistical technique which provides an out of sample estimate for the performance of the classifier when presented with new unseen data^24^. Specifically, we trained the classifier using 203 randomly selected samples, and tested its performance on the remaining 22 samples; the process was repeated 100 times, each time randomly selecting 203 samples for training. The results from the 100 repetitions are summarized in the form mean ± standard deviation. We also present results in the form of a confusion matrix to understand which estimated USV classes are mistakenly confused with which true USV classes (see Supplementary Materials Table 2 for details).

### Projecting the high dimensional feature space in 2D to visualize the differentiation of the USV classes

It is difficult to visualize a high dimensional space (here 102 features), particularly anything beyond three dimensions (3D). Therefore, it would be desirable to have some way of projecting the high dimensional data to something that is intuitively easy to visualize, such as a two-dimensional (2D) space. One method that is widely used in machine learning is called t-distributed Stochastic Neighbor Embedding (t-SNE)^34^. t-SNE is an unsupervised learning approach (that is, the labels are not used) to provide a convenient data visualization framework by projecting the high-dimensional data on a 2D space. It falls under the category of nonlinear dimensionality reduction methods, and it aims to project the data in such a way that the similar samples are close in a 2D scatter plot whereas dissimilar samples are relatively apart. It has strong information theoretic foundations, aiming to minimize a divergence metric between the projected distribution of the samples and the original high dimensional distribution with respect to the projected 2D scatter points. We refer to the study of Maaten and Hinton for further details^34^.

## Supplementary information

Supplementary materials

## Data Availability

The data is available upon request from AV and MLS. The data analysis scripts are available upon request from AT.

## References

[1] Fischer J, Hammerschmidt K (2011). Ultrasonic vocalizations in mouse models for speech and socio-cognitive disorders: insights into the evolution of vocal communication. Genes, Brain and Behavior.

[2] Scattoni M-L, Ricceri L, Crawley JN (2011). Unusual repertoire of vocalizations in adult BTBR T+tf/J mice during three types of social encounters. Genes Brain Behavior.

[3] French CA, Fisher SE (2014). What can mice tell us about Foxp2 function?. Current Opinion in Neurobiology.

[4] Marlin BJ, Mitre M, D’amour JA, Chao MV, Froemke RC (2015). Oxytocin enables maternal behaviour by balancing cortical inhibition. Nature.

[5] Williams WO, Riskin DK, Mott KM (2008). Ultrasonic Sound as an Indicator of Acute Pain in Laboratory Mice. Journal of the American Association for Laboratory Animal Science.

[6] Grant LM (2014). Vocalization deficits in mice over-expressing alpha-synuclein, a model of pre-manifest Parkinson’s disease. Behavioral Neuroscience.

[7] Mo C, Renoir T, Hannan A (2015). Novel ethological endophenotypes in a transgenic mouse model of Huntington’s disease. Behavioural brain research.

[8] Tsai PT (2012). Autistic-like behaviour and cerebellar dysfunction in Purkinje cell Tsc1 mutant mice. Nature.

[9] Burkett, Z. D., Day, N. F., Peñagarikano, O., Geschwind, D. H. & White, S. A. VoICE: A semi-automated pipeline for standardizing vocal analysis across models. *Scientific Reports* **5**, 10.1038/srep10237 (2015). 10.1038/srep10237 PMC4446892 26018425

[10] Holy TE, Guo Z (2005). Ultrasonic Songs of Male Mice. PLoS Biology.

[11] Scattoni M-L, Gandhy SU, Ricceri L, Crawley JN (2008). Unusual Repertoire of Vocalizations in the BTBR T+tf/J Mouse Model of Autism. PLoS ONE.

[12] Branchi, I., Santucci, D., Vitale, A. & Alleva, E. Ultrasonic vocalizations by infant laboratory mice: A preliminary spectrographic characterization under different conditions. *Developmental Psychobiology* **33**, 249–256, doi:10.1002/(SICI)1098-2302(199811)33:3<249::AID-DEV5>3.0.CO;2-R (1998). 10.1002/(sici)1098-2302(199811)33:3<249::aid-dev5>3.0.co;2-r 9810475

[13] Brudzynski, S. M., Kehoe, P. & Callahan, M. Sonographic structure of isolation-induced ultrasonic calls of rat pups. *Developmental Psychobiology* **34**, 195–204, doi:10.1002/(SICI)1098-2302(199904)34:3<195::AID-DEV4>3.0.CO;2-S (1999). 10.1002/(sici)1098-2302(199904)34:3<195::aid-dev4>3.0.co;2-s 10204095

[14] Panksepp JB (2007). Affiliative Behavior, Ultrasonic Communication and Social Reward Are Influenced by Genetic Variation in Adolescent Mice. PLoS ONE.

[15] Fernández-Delgado M, Cernadas E, Barro S, Amorim D (2014). Do we need hundreds of classifiers to solve real world classification problems. Journal of Machine Learning Research.

[16] Tsanas, A. *Ph.D. Thesis*: *Accurate telemonitoring of Parkinson’s disease symptom severity using nonlinear speech signal processing and statistical machine learning*. (Oxford University 2012).

[17] Riede T (2013). Stereotypic Laryngeal and Respiratory Motor Patterns Generate Different Call Types in Rat Ultrasound Vocalization. Journal of experimental zoology. Part A, Ecological genetics and physiology.

[18] Brudzynski, S. M. *Handbook of mammalian vocalization: an integrative neuroscience approach*,. 1st edn, (Academic Press 2009).

[19] Roberts LH (1975). The rodent ultrasound production mechanism. Ultrasonics.

[20] Mahrt E, Agarwal A, Perkel D, Portfors C, Elemans CPH (2016). Mice produce ultrasonic vocalizations by intra-laryngeal planar impinging jets. Current Biology.

[21] Ehret G, Riecke R (2002). Mice and humans perceive multiharmonic communication sounds in the same way. PNAS.

[22] Mundt JC, Vogel AP, Feltner DE, Lenderking WR (2012). Vocal Acoustic Biomarkers of Depression Severity and Treatment Response. Biological Psychiatry.

[23] Romano E, Michetti C, Caruso A, Laviola G, Scattoni M-L (2013). Characterization of Neonatal Vocal and Motor Repertoire of Reelin Mutant Mice. PLoS ONE.

[24] Hastie, T., Tibshirani, R. & Friedman, J. *Unsupervised Learning*. (Springer New York 2009).

[25] Stoppiglia H, Dreyfus G, Dubois R, Oussar Y (2003). Ranking a random feature for variable and feature selection. Journal of machine learning research.

[26] Guyon, I., Gunn, S., Nikravesh, M. & L.A., Z. *Feature Extraction: Foundations and Applications*. 778 (Springer-Verlag 2006).

[27] Hanchuan P, Fuhui L, Ding C (2005). Feature selection based on mutual information criteria of max-dependency, max-relevance, and min-redundancy. IEEE Transactions on Pattern Analysis and Machine Intelligence.

[28] Gilad-Bachrach, R., Navot, A. & Tishby, N. In *Proceedings of the twenty-first international conference on Machine learning* (ed C. Brodley) 43 (ACM, Banff, Alberta, Canada 2004).

[29] Tsanas A, Little MA, Fox C, Ramig LO (2014). Objective Automatic Assessment of Rehabilitative Speech Treatment in Parkinson’s Disease. IEEE Transactions on Neural Systems and Rehabilitation Engineering.

[30] Tsanas A, Little MA, McSharry PE, Spielman J, Ramig LO (2012). Novel Speech Signal Processing Algorithms for High-Accuracy Classification of Parkinson’s Disease. IEEE Transactions on Biomedical Engineering.

[31] Breiman L (2001). Random Forests. Machine Learning.

[32] Cortes C, Vapnik V (1995). Support-vector networks. Machine Learning.

[33] Chih-Wei H, Chih-Jen L (2002). A comparison of methods for multiclass support vector machines. IEEE Transactions on Neural Networks.

[34] Maaten Lvd, Hinton G (2008). Visualizing data using t-SNE. Journal of Machine Learning Research.

