# Supplementary material for: Quantifying ultrasonic mouse vocalizations using acoustic analysis in a supervised statistical machine learning framework
Source: Sci Rep. 2019 May 30;9:8100. doi: 10.1038/s41598-019-44221-3 (PMC6542843; doi:10.1038/s41598-019-44221-3)
Supplement: Supplementary file 1 — Supplementary materials [file 41598_2019_44221_MOESM1_ESM.pdf]

SUPPLEMENTARY MATERIAL

Quantifying ultrasonic mouse vocalizations using acoustic analysis in a supervised statistical machine learning framework

Adam P. Vogel [1,2,3,*,¶] (PhD), Athanasios Tsanas [4,5,¶] (PhD), Maria Luisa Scattoni [6] (PhD)

[1] Centre for Neuroscience of Speech, The University of Melbourne, Victoria, Australia

[2] Department of Neurodegeneration, Hertie Institute for Clinical Brain Research, University of Tübingen, Germany

[3] Redenlab, Australia

[4] Usher Institute of Population Health Sciences and Informatics, Medical School, University of Edinburgh, UK

[5] Oxford Centre for Industrial and Applied Mathematics, Mathematical Institute, University of Oxford, UK

[6] Neurotoxicology and Neuroendocrinology Section, Department of Cell Biology and Neuroscience. Istituto Superiore di Sanità, ROMA Italy

[¶] These authors contributed equally to the work

*Corresponding author:
A/Prof Adam Vogel
Centre for Neuroscience of Speech
The University of Melbourne
550 Swanston Street, Parkville
Melbourne VIC 3010
Australia
Phone: +61 3 9035 5334
Email: vogela@unimelb.edu.au

Supplementary Note I

Automatic Parameter Measurements Setup

The parameters used to generate spectrograms are described in Supplementary Figure 1.

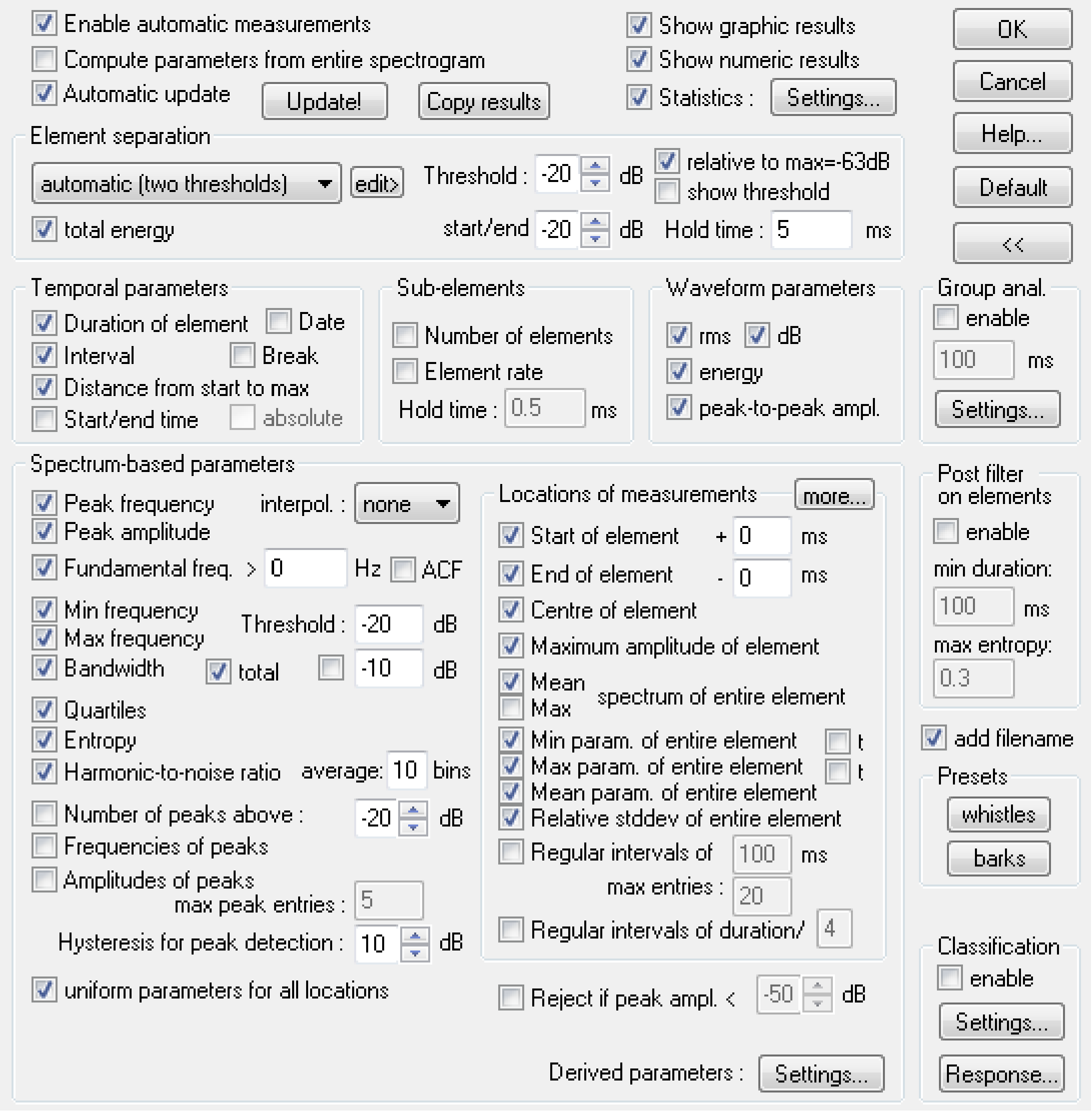

Supplementary Figure 1: Automatic Parameter Measurements Setup

Acoustic measures derived from Avisoft included summative (i.e. mean/entire) and position specific (i.e. start/end of call) measures of duration (e.g. length of call), frequency (maximum frequency), amplitude (standard deviation intensity), quality (e.g. noise to harmonics ratio), and entropy (i.e. disorder or randomness) at different positions along the call (see Supplementary Table 1 for complete list).

Supplementary Table 1: Acoustic measures applied to USVs (see Avisoft SASLab manual for more detail)

TEMPORAL PARAMETERS: Duration, interval, distance from start to max.
WAVEFORM PARAMETERS: Root mean squared, energy, peak to peak amplitude.
SPECTRUM BASED PARAMETERS: peak frequency (start), peak amplitude (start), fundamental frequency (start), minimum frequency (start), maximum frequency (start), bandwidth (start), 25th quartile (start), 50th quartile (start), 75th quartile (start), entropy (start), harmonics to noise ratio (start), peak frequency (end), peak amplitude (end), fundamental frequency (end), minimum frequency (end), maximum frequency (end), bandwidth (end), 25th quartile (end), 50th quartile (end), 75th quartile (end), entropy (end), harmonics to noise ratio (end), peak frequency (centre), peak amplitude (centre), fundamental (centre), minimum frequency (centre), maximum frequency (centre), bandwidth (centre), 25th quartile (centre), 50th quartile (centre), 75th quartile (centre), entropy (centre), harmonics to noise ratio (centre), peak frequency (maximum), peak amplitude (maximum), fundamental (maximum), minimum frequency (maximum), maximum frequency (maximum), bandwidth (maximum), 25th quartile (maximum), 50th quartile (maximum), 75th quartile (maximum), entropy (maximum), harmonics to noise ratio (maximum), peak frequency (mean), peak amplitude (mean), fundamental (mean), minimum frequency (mean), maximum frequency (mean), bandwidth (mean), 25th quartile (mean), 50th quartile (mean), 75th quartile (mean), entropy (mean), harmonics to noise ratio (mean), peak frequency (minimum entire), peak amplitude (minimum entire), fundamental (minimum entire), minimum frequency (minimum entire), maximum frequency (minimum entire), bandwidth (minimum entire), 25th quartile (minimum entire), 50th quartile (minimum entire), 75th quartile (minimum entire), entropy (minimum entire), harmonics to noise ratio (minimum entire), peak frequency (maximum entire), peak amplitude (maximum entire), fundamental (maximum entire), minimum frequency (maximum entire), maximum frequency (maximum entire), bandwidth (maximum entire), 25th quartile (maximum entire), 50th quartile (maximum entire), 75th quartile (maximum entire), entropy (maximum entire), harmonics to noise ratio (maximum entire), peak frequency (mean entire), peak amplitude (mean entire), fundamental (mean entire), minimum frequency (mean entire), maximum frequency (mean entire), bandwidth (mean entire), 25th quartile (mean entire), 50th quartile (mean entire), 75th quartile (mean entire), entropy (mean entire), harmonics to noise ratio (mean entire), peak frequency (standard deviation entire), peak amplitude (standard deviation entire), fundamental (standard deviation entire), minimum frequency (standard deviation entire), maximum frequency (standard deviation entire), bandwidth (standard deviation entire), 25th quartile (standard deviation entire), 50th quartile (standard deviation entire), 75th quartile (standard deviation entire), entropy (standard deviation entire), harmonics to noise ratio (standard deviation entire).

Supplementary Note II: Additional results

Presenting algorithmic performance in terms of a confusion matrix

In the main manuscript, we provided the summarized classification performance on out-of-sample data in the form of accuracy. However, it is useful to understand how class membership is confused, i.e. which class is confused by which. Here, we provide the confusion matrix for the results when the RF was presented with the top eight features selected using SIMBA.

Supplementary Materials Table 2: Confusion matrix summarizing the out-of-sample classification performance for the 9 USV classes, when RF is fed with the top eight features selected using SIMBA.

		**Estimated class membership using the RF**								
True class membership	'Chevron'	'Chevron'	'Complex'	'Composite'	'Downward'	'Flat'	'Frequency step'	'Short'	'Two components'	'Upward'
	'Comple	**156**	13	0	33	0	0	24	0	11
	'Compos	23	**202**	0	46	0	0	0	0	7
	'Downw	0	1	**152**	5	6	14	0	24	2
	'Flat'	38	10	8	**188**	0	0	0	0	3
	'Frequen	4	0	5	0	**235**	0	0	0	0

	'Short'	0	0	5	0	0	**222**	0	17	0
	'Two	0	0	0	0	12	0	**245**	0	0
	'Upward'	0	0	14	0	0	1	0	**217**	0
	'Chevron	17	0	0	1	6	0	0	0	**233**

The total sum in the table is 2200 out-of-sample estimates, reflecting the 100 repetitions where in each repetition 90% of the data was used for training (203 out of the 225 samples), and 10% for testing (22 out of the 225 samples). An a_{ij} entry in the table reflects the number of samples which truly belong in class *i*, which were classified as belonging in class *j*. A perfect accuracy model would have all entries in the long diagonal and zero otherwise.

Overall, the results in Supplementary Materials Table S2 demonstrate that all USV classes are accurately estimated. We remark that particularly the classes "Frequency step", "Short" and "Two components" are very accurately estimated using the eight selected features; "Chevron" and "Downward" appear to be somewhat less accurately estimated.

Supplementary Note III: Evidence that Scattoni et al. [1] is a standard approach for call categorization.

The following studies have utilized the same call categories as Scattoni (M.L. Scattoni, S.U. Gandhy, L. Ricceri, J.N. Crawley. Unusual repertoire of vocalizations in the BTBR T+tf/J mouse model of autism. PLoS One. 2008 Aug 27;3(8):e3067[1].

1. M.L. Scattoni, L. Ricceri, J.N. Crawley, Unusual repertoire of vocalizations in adult BTBR T+tf/J mice during three types of social encounters. Genes, brain, and behavior. 2011 Feb;10(1):44-56[2].

2. E.R. Fraley, Z.D. Burkett, N.F. Day, B.A. Schwartz, P.E. Phelps, S.A. White. Mice with Dab1 or Vldlr insufficiency exhibit abnormal neonatal vocalization patterns. Scientific Report. 2016 May 17;6:25807[3].

3. S.L. Hodges, S.O. Nolan, C.D. Reynolds, J.N. Lugo, 2017. Spectral and temporal properties of calls reveal deficits in ultrasonic vocalizations of adult Fmr1 knockout mice. Behavioural Brain Research. 2017 May 26;332:50-58[4].

4. S. Roy, N. Watkins, D. Heck. Comprehensive analysis of ultrasonic vocalizations in a mouse model of fragile x syndrome reveals limited, call type specific deficits. PLoS One. 2012;7(9):e44816[5]

5. E. Romano, C. Michetti, A. Caruso, G. Laviola, M.L. Scattoni. Characterization of neonatal vocal and motor repertoire of reelin mutant mice. PLoS One. 2013 May 21;8(5):e64407[6].

6. C. Michetti, E. Romano, L. Altabella, A. Caruso, P. Castelluccio, G. Bedse, S. Gaetani, R. Canese, G. Laviola, M.L. Scattoni. Mapping pathological phenotypes in reelin mutant mice. Frontiers in Pediatrics. 2014 Sep 4;2:95[7].

7. Moldrich, R. X., Leanage, G., She, D., Dolan-Evans, E., Nelson, M., Reza, N., and Reutens, D. C. (2013). "Inhibition of histone deacetylase in utero causes sociability deficits in postnatal mice," Behavioural Brain Research 257, 253-264[8].

8. Hiramoto, T., Kang, G., Suzuki, G., Satoh, Y., Kucherlapati, R., Watanabe, Y., and Hiroi, N. (2011). "Tbx1: identification of a 22q11.2 gene as a risk factor for autism spectrum disorder in a mouse model," Human Molecular Genetics 20, 4775-4785[9].

9. Reynolds, C. D., Nolan, S. O., Huebschman, J. L., Hodges, S. L., and Lugo, J. N. (2017). "Early-life status epilepticus acutely impacts select quantitative and qualitative features of neonatal vocalization behavior: Spectrographic and temporal characterizations in C57BL/6 mice," Epilepsy & Behavior 72, 58-62[10].

Adapted from Scattoni et al., [1]

1) Kikusui, T., Nakanishi, K., Nakagawa, R., Nagasawa, M., Mogi, K., and Okanoya, K. (2011). "Cross Fostering Experiments Suggest That Mice Songs Are Innate," PLOS ONE 6,

e17721 [11]. According to previous studies (Scattoni et al 2008), each syllable was identified as 1 of 10 distinct categories: “upward,” “flat,” “chevron,” “complex,” “more jumps,” “downwards,” “short,” “wave,” “one jump,” or “harmonics”.

2) Chabout, J., Serreau, P., Ey, E., Bellier, L., Aubin, T., Bourgeron, T., and Granon, S. (2012). "Adult Male Mice Emit Context-Specific Ultrasonic Vocalizations That Are Modulated by Prior Isolation or Group Rearing Environment," PLOS ONE 7, e29401. [12]. Adapted from Scattoni et al 2008, the ten categories were:

1. Short: ≤50 ms and ≤10 kHz frequency modulation.
2. Flat: ≥50 ms and ≤10 kHz frequency modulation.
3. One frequency jump: instantaneous frequency step, like a vertical discontinuity with no time gap.
4. Multiple frequency jumps: multiple instantaneous frequency step.
5. U: U-shape wave ≥10 kHz frequency modulation.
6. Chevron: inverted-U shape ≥10 kHz frequency modulation.
7. Modulated: ≥10 kHz of modulation, several decreases and increases in frequency.
8. Composite: two or more components emitted simultaneously.
9. Upward: continuous increase in peak frequency ≥10 kHz frequency modulation.
10. Downward: continuous decrease in peak frequency ≥10 kHz frequency modulation

3) Hanson, J. L., and Hurley, L. M. (2012). "Female Presence and Estrous State Influence Mouse Ultrasonic Courtship Vocalizations," PLOS ONE 7, e40782[13]. Adapted from previously described methods Scattoni et al 2008 and Grimsley 2011:

- Short syllables were less than 10 ms in duration.
- Flat syllables had less than 5 kHz of modulation.
- Harmonic syllables contained at least one segment with at least one harmonic (most of these also had breaks in frequency).

- Jump syllables contained at least one break in frequency with no break in intensity (and no harmonics).

- Up syllables increased in frequency (sweep>5 kHz).

- Down syllables decreased in frequency (sweep>5 kHz).

- Arc syllables increased and then decreased in frequency, with the highest frequency reaching >5 kHz above the beginning and end frequencies.

- U syllables decreased and then increased in frequency, with the lowest frequency reaching >5 kHz below the beginning and end frequencies.

- Complex syllables contained two or more directional changes in frequency and >5 kHz modulation of frequency.

4) Nakagawa, R., Matsunaga, E., and Okanoya, K. (2012). "Defects in Ultrasonic Vocalization of Cadherin-6 Knockout Mice," PLOS ONE 7, e49233[14]. Each call is categorized as the 1 of 10 distinct categories, based on internal pitch change, length, and shape, according to previously reported categories with minor modifications (Scattoni et al 2008): Cheveron", "Complex", "Downward", "Flat", "Short", "Upward", "Wave", "Harmonics", "One jump", "More jumps".

5) Lopatina, O. L., Furuhara, K., Ishihara, K., Salmina, A. B., and Higashida, H. (2017). "Communication Impairment in Ultrasonic Vocal Repertoire during the Suckling Period of Cd157 Knockout Mice: Transient Improvement by Oxytocin," Frontiers in Neuroscience 11, 266[15]. Each syllable was classified as one of the following seven waveform categories: upward (up), downward (down), chevron, complex, harmonic, plate, or "V"-call. Classification was determined by internal pitch change, length, and shape, and was based on and adapted from previously described methods (Scattoni et al., 2008; Grimsley et al., 2011; Liu et al., 2013a; Zampieri et al., 2014)

6) Grimsley, J. M. S., Monaghan, J. J. M., and Wenstrup, J. J. (2011). "Development of Social Vocalizations in Mice," PLOS ONE 6, e17460[16]. We identified 11 syllable types, 9 of which were similar to those described by Scattoni et al. 2008. In contrast to their study,

however, we did not classify harmonic (termed composite by Scattoni et al., 2008) or nonlinear (termed harmonic by Scattoni et al., 2008).

7) Z.D. Burkett, N.F. Day, O. Peñagarikano, D.H. Geschwind, S.A. White. VoICE: A semi-automated pipeline for standardizing vocal analysis across models. Scientific Reports. 2015 May 28;5:10237[17]. Individual calls were classified into the canonical types as described by Scattoni et al. 2008 and we added categories called "double" and "triple" and a "miscellaneous"

8) E. Ey, N. Torquet, A.M. Le Sourd, C.S. Leblond, T.M. Boeckers, P. Faure, T. Bourgeron, The Autism ProSAP1/Shank2 mouse model displays quantitative and structural abnormalities in ultrasonic vocalisations. Behavioural Brain Research. 2013 Nov 1;256:677-89[18]. Each call was classified manually in one call type over five ("short", "simple", "complex", "unstructured", and "with frequency jumps"). These call types regrouped call subtypes from Schmeisser et al 2012 based on Scattoni et al 2008

9) Schmeisser MJ, Ey E, Wegener S, Bockmann J, Stempel AV, Kuebler A, Janssen AL, Udvardi PT, Shiban E, Spilker C, Balschun D, Skryabin BV, Dieck St, Smalla KH, Montag D, Leblond CS, Faure P, Torquet N, Le Sourd AM, Toro R, Grabrucker AM, Shoichet SA, Schmitz D, Kreutz MR, Bourgeron T, Gundelfinger ED, Boeckers TMAutistic-like behaviours and hyperactivity in mice lacking ProSAP1/Shank2. Nature. 2012 Apr 29;486(7402):256-60[19]. Each call was classified in one call category of 11, adapted from Scattoni et al. 2008.

11) Yang, M., Loureiro, D., Kalikhman, D., and Crawley, J. N. (2013). "Male mice emit distinct ultrasonic vocalizations when the female leaves the social interaction arena," Frontiers in Behavioral Neuroscience 7, 159[20]. Calls were classified into eight categories, generally based on criteria described previously (Scattoni et al., 2011). Categories analyzed in this study are: complex, two-component, upward, downward, chevron, short, frequency steps, and flat. "Unstructured" and "Composite" described in a previous study (Scattoni et al., 2011) were rarely detected in our study, and were not analyzed.

12) Sugimoto H, Okabe S, Kato M, Koshida N, Shiroishi T, Mogi K, Kikusui T, Koide T. A role for strain differences in waveforms of ultrasonic vocalizations during male-female interaction. PLoS One. 2011;6(7):e22093[21]. We categorized the waveforms into nine types,

which is consistent with the results described in a recent report Scattoni et al 2008, with minor modification (Flat, Jump, Short, Upward, A-Type, U-Type, Harmonic, Downward, Complex).

13) Heckman, J., McGuinness, B., Celikel, T., and Englitz, B. (2016). "Determinants of the mouse ultrasonic vocal structure and repertoire," Neuroscience & Biobehavioral Reviews 65, 313-325[22]. No universally accepted classification or method has emerged so far. In order to address syllable types in this review, we adopt the classification method from Scattoni et al. (2008) and (Grimsley et al., 2011) with the exception of the Low Frequency Harmonic syllables, as they are (for the most part) not ultrasonic.

14) Miranda, R., Nagapin, F., Bozon, B., Laroche, S., Aubin, T., and Vaillend, C. (2015). "Altered social behavior and ultrasonic communication in the dystrophin-deficient mdx mouse model of Duchenne muscular dystrophy," Molecular Autism 6, 60 [23] Identification of 10 waveform pattern categories (Scattoni 2008; Chabout 2012; Ey 2013): upward: continuous frequency increase of at least 1.5 Hz per 10-ms bins, with eventually flat steps; downward: continuous frequency decrease of at least 1.5 Hz per 10-ms bins, with eventually flat steps; peak: frequency-modulated call showing continuous increase in frequency followed by a continuous decrease; u-shape: frequency-modulated call showing continuous decrease in frequency followed by a continuous increase; flat: constant frequency with no modulation >1.5 Hz per 10-ms bins; short: duration <10 ms; sinusoidal (complex): two or more directional changes in frequency in distinct directions; frequency jump (composite): two or more components displaying discontinuous frequency “jump (s)” on the sonographic representation but without gap on the time scale; harmonic: fundamental frequency slightly modulated combined with an amplified harmonic component. Because harmonics were observed in different call categories, each call was first assigned to one of the above categories and then further labeled as “harmonic” or “non-harmonic”; unstructured: without main sound component and frequency shape that could be assimilated to any of the other categories. Calls were then grouped in a reduced number of broader (inclusive) categories defined as: simple (i.e., upward, downward, flat, short, peak, and u-shaped), complex (i.e., sinusoidal), and composite (i.e., frequency jump).

15) Zampieri, B. L., Fernandez, F., Pearson, J. N., Stasko, M. R., and Costa, A. C. S. (2014). "Ultrasonic vocalizations during male–female interaction in the mouse model of Down syndrome Ts65Dn," Physiology & Behavior 128, 119-125[24]. Each syllable was classified as

one of 9 waveform categories based on internal pitch change, length and shape, according to previously reported nomenclature (Scattoni et al 2008). The syllable categories are described below: Arc syllables (also referred to as Chevron syllables) waveforms with increases and then decreases in frequency, with the highest frequency reaching N 5 kHz above the beginning and end frequencies. Down syllables (also referred to as Downward syllables) waveforms exhibiting a continual decrease in frequency, with a sweep N 5 kHz. Flat syllables waveforms with less than 5 kHz of modulation. Jump syllables (also referred to as One-Jump syllables) waveforms containing one break in frequency, but without breaks in intensity. Short syllables waveforms less than 10 ms in duration. U syllables waveforms exhibiting decreases and then increases in frequency, with the lowest frequency reaching N 5 kHz below the beginning and end frequencies. Upsyllables (also referred to as Upward syllables) waveforms showing a continual uniform increase in frequency, with a sweep N 5 kHz. Complex syllables waveforms containing two or more directional changes in frequency and N 5 kHz modulation. Multiple jumps syllables waveforms containing at least two breaks in frequency with no break in intensity

Other/different Categorizations from Scattoni et al., 2008

1) Grimsley, J. M. S., Sheth, S., Vallabh, N., Grimsley, C. A., Bhattal, J., Latsko, M., Jasnow, A., and Wenstrup, J. J. (2016). "Contextual Modulation of Vocal Behavior in Mouse: Newly Identified 12 kHz “Mid-Frequency” Vocalization Emitted during Restraint," Frontiers in Behavioral Neuroscience 10, 38[25]. USVs were separated into two subtypes based on the presence or absence of an abrupt frequency step. Tonal USVs (tUSV) do not have abrupt frequency steps, while stepped USVs (stpUSV) have one or more abrupt steps of ≥10 kHz.

2) Keesom, S. M., Finton, C. J., Sell, G. L., and Hurley, L. M. (**2017**). "Early-Life Social Isolation Influences Mouse Ultrasonic Vocalizations during Male-Male Social Encounters," PLOS ONE **12**, e0169705 [26]. We used frequency and the presence of frequency jumps to categorize USVs into four types, defined as follows: “70-kHz plain” (lowest frequency at 70 kHz and no frequency jumps present), “70-kHz jump” (lowest frequency at 70 kHz and jumps in frequency present), “50-kHz plain” (lowest frequency at 50 kHz and harmonic frequency at 100 kHz), and “50-kHz jump” (lowest frequency at 50 kHz, harmonic frequency at 100 kHz, and jumps in frequency present).

3) Wang, H., Liang, S., Burgdorf, J., Wess, J., and Yeomans, J. (**2008**). "Ultrasonic Vocalizations Induced by Sex and Amphetamine in M2, M4, M5 Muscarinic and D2 Dopamine Receptor Knockout Mice," PLOS ONE **3**, e1893[27]. The duration, peak frequency and bandwidth of USVs were analyzed according to the Avisoft software manual. The USVs with bandwidth ≤5-kHz is considered as flat USVs, whereas those whose bandwidth being >5-kHz is considered as frequency-modulated USVs.

4) Gaub, S., Fisher, S. E., and Ehret, G. (**2016**). "Ultrasonic vocalizations of adult male Foxp2-mutant mice: behavioral contexts of arousal and emotion," Genes, Brain and Behavior **15**, 243-259 [28]. Division of USVs in simple calls without frequency jumps and calls with frequency jumps using the terminology of jump types according to Holy and Guo (2005), as jump types h, d, u, hd, hdu, du, other types, and expanded by the two further jump types hh and uh

5) Holy, T. E., and Guo, Z. (2005). "Ultrasonic Songs of Male Mice," PLoS Biology 3, e386. [29]. We grouped syllables into only two categories, depending on whether they did (“1”) or did not (“0”) contain one or more low jumps.

6) Musolf, K., Meindl, S., Larsen, A. L., Kalcounis-Rueppell, M. C., and Penn, D. J. (2015). "Ultrasonic Vocalizations of Male Mice Differ among Species and Females Show Assortative Preferences for Male Calls," PLOS ONE 10, e0134123 [30]. The syllables produced by each male were analyzed in detail, and based on characteristic spectrographic shape and parameters, syllables were classified into 7 categories as follows: (1) Frequency Upsweep: continuous increase in peak frequency ≥ 10kHz frequency modulation; (2) Frequency Downsweep: continuous decrease in peak frequency ≥ 5kHz frequency modulation; (3) Constant Modulated: ≤ 2kHz frequency modulation; (4) U-Shaped: U-shape wave ≥ 4kHz frequency modulation; (5) U-Shaped Inverted (Chevron): inverted-U shape ≥ 4kHz frequency modulation; (6) 1-Frequency-Step: instantaneous frequency step, like a vertical discontinuity with no time gap; (7) 2-Frequency-Step: 2 instantaneous frequency steps.

References

1. Scattoni, M.-L., Gandhy, S.U., Ricceri, L. & Crawley, J.N. Unusual Repertoire of Vocalizations in the BTBR T+tf/J Mouse Model of Autism. *PLoS ONE* **3**, e3067 (2008).
2. Scattoni, M.-L., Crawley, J.N. & Ricceri, L. Ultrasonic vocalizations: A tool for behavioural phenotyping of mouse models of neurodevelopmental disorders. *Neuroscience & Biobehavioral Reviews* **33**, 508-515 (2009).
3. Fraley, E.R. et al. Mice with Dab1 or Vldlr insufficiency exhibit abnormal neonatal vocalization patterns. *Scientific Reports* **6**, 25807 (2016).
4. Hodges, S.L., Nolan, S.O., Reynolds, C.D. & Lugo, J.N. Spectral and temporal properties of calls reveal deficits in ultrasonic vocalizations of adult Fmr1 knockout mice. *Behavioural Brain Research* **332**, 50-58 (2017).
5. Roy, S., Watkins, N. & Heck, D. Comprehensive Analysis of Ultrasonic Vocalizations in a Mouse Model of Fragile X Syndrome Reveals Limited, Call Type Specific Deficits. *PLOS ONE* **7**, e44816 (2012).
6. Romano, E., Michetti, C., Caruso, A., Laviola, G. & Scattoni, M.-L. Characterization of Neonatal Vocal and Motor Repertoire of Reelin Mutant Mice. *PLoS ONE* **8**, e64407 (2013).
7. Michetti, C. et al. Mapping Pathological Phenotypes in Reelin Mutant Mice. *Frontiers in Pediatrics* **2** (2014).
8. Moldrich, R.X. et al. Inhibition of histone deacetylase in utero causes sociability deficits in postnatal mice. *Behavioural Brain Research* **257**, 253-264 (2013).
9. Hiramoto, T. et al. Tbx1: identification of a 22q11.2 gene as a risk factor for autism spectrum disorder in a mouse model. *Human Molecular Genetics* **20**, 4775-4785 (2011).
10. Reynolds, C.D., Nolan, S.O., Huebschman, J.L., Hodges, S.L. & Lugo, J.N. Early-life status epilepticus acutely impacts select quantitative and qualitative features of neonatal vocalization behavior: Spectrographic and temporal characterizations in C57BL/6 mice. *Epilepsy & Behavior* **72**, 58-62 (2017).
11. Kikusui, T. et al. Cross Fostering Experiments Suggest That Mice Songs Are Innate. *PLOS ONE* **6**, e17721 (2011).
12. Chabout, J. et al. Adult Male Mice Emit Context-Specific Ultrasonic Vocalizations That Are Modulated by Prior Isolation or Group Rearing Environment. *PLOS ONE* **7**, e29401 (2012).
13. Hanson, J.L. & Hurley, L.M. Female Presence and Estrous State Influence Mouse Ultrasonic Courtship Vocalizations. *PLOS ONE* **7**, e40782 (2012).
14. Nakagawa, R., Matsunaga, E. & Okanoya, K. Defects in Ultrasonic Vocalization of Cadherin-6 Knockout Mice. *PLOS ONE* **7**, e49233 (2012).
15. Lopatina, O.L., Furuhara, K., Ishihara, K., Salmina, A.B. & Higashida, H. Communication Impairment in Ultrasonic Vocal Repertoire during the Suckling Period of Cd157 Knockout Mice: Transient Improvement by Oxytocin. *Frontiers in Neuroscience* **11**, 266 (2017).
16. Grimsley, J.M.S., Monaghan, J.J.M. & Wenstrup, J.J. Development of Social Vocalizations in Mice. *PLOS ONE* **6**, e17460 (2011).
17. Burkett, Z.D., Day, N.F., Peñagarikano, O., Geschwind, D.H. & White, S.A. VoICE: A semi-automated pipeline for standardizing vocal analysis across models. *Scientific Reports* **5** (2015).

18. Ey, E. et al. The Autism ProSAP1/Shank2 mouse model displays quantitative and structural abnormalities in ultrasonic vocalisations. *Behavioural Brain Research* **256**, 677-689 (2013).
19. Schmeisser, M.J. et al. Autistic-like behaviours and hyperactivity in mice lacking ProSAP1/Shank2. *Nature* **486**, 256-260 (2012).
20. Yang, M., Loureiro, D., Kalikhman, D. & Crawley, J.N. Male mice emit distinct ultrasonic vocalizations when the female leaves the social interaction arena. *Frontiers in Behavioral Neuroscience* **7**, 159 (2013).
21. Sugimoto, H. et al. A Role for Strain Differences in Waveforms of Ultrasonic Vocalizations during Male–Female Interaction. *PLOS ONE* **6**, e22093 (2011).
22. Heckman, J., McGuinness, B., Celikel, T. & Englitz, B. Determinants of the mouse ultrasonic vocal structure and repertoire. *Neuroscience & Biobehavioral Reviews* **65**, 313-325 (2016).
23. Miranda, R. et al. Altered social behavior and ultrasonic communication in the dystrophin-deficient mdx mouse model of Duchenne muscular dystrophy. *Molecular Autism* **6**, 60 (2015).
24. Zampieri, B.L., Fernandez, F., Pearson, J.N., Stasko, M.R. & Costa, A.C.S. Ultrasonic vocalizations during male–female interaction in the mouse model of Down syndrome Ts65Dn. *Physiology & Behavior* **128**, 119-125 (2014).
25. Grimsley, J.M.S. et al. Contextual Modulation of Vocal Behavior in Mouse: Newly Identified 12 kHz "Mid-Frequency" Vocalization Emitted during Restraint. *Frontiers in Behavioral Neuroscience* **10**, 38 (2016).
26. Keesom, S.M., Finton, C.J., Sell, G.L. & Hurley, L.M. Early-Life Social Isolation Influences Mouse Ultrasonic Vocalizations during Male-Male Social Encounters. *PLOS ONE* **12**, e0169705 (2017).
27. Wang, H., Liang, S., Burgdorf, J., Wess, J. & Yeomans, J. Ultrasonic Vocalizations Induced by Sex and Amphetamine in M2, M4, M5 Muscarinic and D2 Dopamine Receptor Knockout Mice. *PLOS ONE* **3**, e1893 (2008).
28. Gaub, S., Fisher, S.E. & Ehret, G. Ultrasonic vocalizations of adult male Foxp2-mutant mice: behavioral contexts of arousal and emotion. *Genes, Brain and Behavior* **15**, 243-259 (2016).
29. Holy, T.E. & Guo, Z. Ultrasonic Songs of Male Mice. *PLoS Biology* **3**, e386 (2005).
30. Musolf, K., Meindl, S., Larsen, A.L., Kalcounis-Rueppell, M.C. & Penn, D.J. Ultrasonic Vocalizations of Male Mice Differ among Species and Females Show Assortative Preferences for Male Calls. *PLOS ONE* **10**, e0134123 (2015).
